# Acute Effects of Exogenous Hormone Administration on Postprandial Acylation Stimulating Protein Levels in Ovariectomized Rats after a Fat Load

**DOI:** 10.1155/2014/510916

**Published:** 2014-12-02

**Authors:** Bashair Al Riyami, Marah El-Tahir, Sultan Al Maskari, Eugene H. Johnson, Jumana Saleh

**Affiliations:** ^1^Department of Biochemistry, College of Medicine, Sultan Qaboos University, Al-Khod, 123 Muscat, Oman; ^2^Department of Microbiology and Immunology, College of Medicine, Sultan Qaboos University, Al-Khod, 123 Muscat, Oman; ^3^Department of Animal and Veterinary Sciences, College of Agricultural and Marine Sciences, Sultan Qaboos University, Al-Khod, 123 Muscat, Oman

## Abstract

*Background*. ASP, a potent lipogenic factor, was linked to female fat metabolism in association studies. *Aim*. To investigate acute effects of sex hormone treatment on postprandial ASP levels *in vivo*. *Methods*. 24 female rats were randomly divided into 4 groups including controls. The rats were ovariectomized and injected with progesterone, estrogen, or testosterone. An hour later, olive oil was administered orally. Plasma ASP and triglycerides were measured at several postprandial time points. Area under the curve (TG-AUC) represented TG clearance. *Results*. Only the progesterone treated group had a significant postprandial ASP increase at two hours compared to basal levels (439.8 ± 62.4 versus 253.4 ± 59.03 *μ*g/mL, *P* = 0.04). Interestingly, increased ASP levels coordinated negatively with corresponding TG levels and TG-AUC postprandially, mostly evident in the opposite effects in the progesterone and testosterone treated groups. ASP levels increased 3-fold in the progesterone versus testosterone treated groups, whereas TG-AUC was significantly lower. *Conclusion*. These findings suggest that progesterone enhances ASP production and TG clearance simultaneously, supporting the notion of a stimulatory role for progesterone on ASP-mediated TG clearance. This is the first functional study demonstrating a cause-effect relationship between hormone treatment and ASP levels *in vivo* and may contribute to understanding the mechanism of progesterone function as a female lipogenic hormone.

## 1. Introduction

Acylation stimulating protein (ASP) is a small basic protein that consists of 76-amino acids produced from adipocytes. ASP is produced by the interaction of complement C3, factor B, and adipsin produced in adipose tissue [1]. Despite numerous identified adipose tissue derived factors, ASP uniquely demonstrated a great effect in lipogenesis stimulation. ASP actively stimulates TG synthesis in human fibroblasts and to a much greater extent in adipocytes [2]. This occurs through the enhanced esterification of fatty acids in adipose tissue by increasing the activity of diacylglycerol acyltransferase (DGAT), the rate limiting enzyme in TG synthesis, and indirectly enhances the activity of lipoprotein lipase (LPL) in adipocytes by increasing fatty acid trapping within adipocytes [3]. ASP was also found to inhibit hormone-sensitive lipase (HSL) [4]. Interestingly, ASP was shown to be as potent as insulin in its fat storage effects. However, ASP effects were independent, and additive, to insulin. Dietary triglyceride rich lipoproteins, chylomicrons (CHYLO) were the most potent stimulators of ASP production* in vitro* [5]. In animal studies, using a C3 knockout (ASP deficient) mice showed delayed postprandial TG clearance and reduced TG storage capacity after a fat load [2, 6], which highlighted the important role of ASP in the postprandial TG clearance. Such an effect was diminished by the administration of exogenous ASP accelerating the clearance of free fatty acids and TG from the circulation after oral fat administration [2]. ASP treatment stimulated weight gain and decreased energy expenditure in the mice [7]. In humans, after a fat load, the production of ASP increased postprandially in correlation with plasma TG clearance [8]. Adipose metabolism is mediated through a complex network of hormonal signals including sex hormones [9]. The relationship between sex hormones and ASP production and function is not yet understood. Extensive studies exploring the role of sex hormones in rodents and humans have demonstrated the involvement of sex hormones in the modulation of adipose tissue metabolism through lipolytic and lipogenic pathways. Estrogen has been described as a lipolytic hormone [10], while progesterone was shown to enhance fat storage and exhibit lipogenic effects [11]. The role of sex hormones in fat distribution remains poorly understood and it is mainly attributed to their effects on lipogenic hormones, transcription factors, and enzymes that act together to determine the outcome. The effects of sex hormones on fat storage factors, such as insulin, have been investigated in several studies; however, no clear connection has been established due to conflicting evidence [12]. Recent studies have shown novel findings linking ASP to fat metabolism and hormonal changes in females. ASP was found to strongly associate with fluctuating progesterone levels in females across the menstrual cycle whereas no association was found with insulin [13]. At late gestation, ASP levels increased significantly along with natural increases in female hormones, particularly progesterone which increases late during pregnancy, whereas maternal triglyceride levels also strongly correlated with fetal birth weight [14]. Interestingly, also cord blood ASP levels correlated positively with fetal birth weight [15]. In addition, elevations in ASP levels were seen in women with metabolic diseases that included hormonal disturbances such as polycystic ovary syndrome (PCOS) [16, 17], suggesting that ASP metabolism may be altered in association with sex hormones disturbances. The connection of ASP to female fat metabolism and hormonal changes is collated in a recent review [18]. Although associations of ASP levels with female hormone alterations have been reported, a cause-effect relationship of sex hormone and ASP production and function has not been established. Therefore, animal studies investigating the direct role of sex hormones on the function of ASP in postprandial fat clearance may be of significant importance in this regard. In this study, we investigated the effect of exogenous hormone administration (of different sex hormones) on postprandial ASP and TG after a fat load in ovariectomized rats.

## 2. Materials and Methods

### 2.1. Animals

All animal experiments carried out in this study were handled and conducted in the small animals' house and approved by the Animal Ethics Committee at Sultan Qaboos University. The animals were housed in a temperature controlled room (23°C) with a 12 : 12 light : dark cycle and free access to food and water.

### 2.2. Experimental Design

24 female Wister rats (8 weeks of age) were randomly divided into 4 groups (*n* = 6). All rats were ovariectomized (OVX). On day 14 after the OVX procedure, a postprandial experiment was conducted under different hormone treatments. The concentrations of ovarian steroids were expected to reach nadir within 10 to 23 days after the OVX procedure [19], which was confirmed before running the main experiment on a different set of rats.

### 2.3. Surgical Procedure

At age of 8 weeks, rats were ovariectomized under general anesthesia using ketamine/xylazine combination, dosed at 75 mg/kg body weight and 6 mg/kg body weight. The ovariectomy procedure was conducted as described by Waynforth and Flecnell [20]; briefly, a small midline dorsal skin incision (1-2 cm) was made halfway between the caudal edge of the ribcage and the base of the tail. A small cut then was made into the muscle wall and the ovary was gently pulled and a ligature was placed below the ovary, and the ovary was then removed. The remaining tissue was replaced into the peritoneal cavity. Muscle layer and skin were sutured. The ovary on the other side is removed in a similar manner.

### 2.4. Hormone Treatments

After surgery, ovariectomized rats were allowed to recover from surgery stress for 14 days. All hormones were purchased from Sigma Aldrich. All hormones were delivered by sesame oil subcutaneously. The progesterone and estrogen doses used in this experiment were modified to maintain acceptable high physiological levels that mimic late gestation pregnancy in rats (Table 1) [21–24].

### 2.5. Postprandial Fat Clearance

Fourteen days after being ovariectomized, fasting rats were injected subcutaneously with single dose of different hormone treatments delivered in 0.2 mL sesame oil. A control group received sesame oil injection only. One hour later, a fat load consisting of 1.5 mL olive oil was administered by oral gavage [25, 26]. Plasma blood samples were collected at basal levels (time 0) followed by 2, 4, 6, and 8 hours after oil administration. Blood was collected into EDTA containing tubes, centrifuged at 2000 rpm for 10 minutes at 4°C. Plasma was isolated and stored at −80°C until analysis.

### 2.6. Analysis

Plasma ASP was measured in plasma using rat C3a ELISA (Novatein Biosciences Inc., USA). TG levels were measured by enzymatic colorimetric method using commercial assay kits (Roche) and automated* cobas 111c analyzer* (Roche). All protocols were conducted according to the kits manufacturer's instruction.

### 2.7. Statistical Analysis

Differences in ASP and TG baseline levels and the weights of rats (controls and hormone treated groups) were compared by one-way ANOVA followed by Bonferroni post analysis based on the assumption of homogeneity of variances for the tested groups. Tamhane's T2 post hoc analysis was used when the assumption of equal variances was not met. To evaluate the effect of hormonal intervention on ASP level changes over the postprandial period, two-way repeated measures analysis of covariance (2-way ANCOVA) was performed for comparisons in ASP levels between the hormone treated groups after adjusting for weight as a covariate. The required assumption of sphericity was ensured by Mauchly's test. Greenhouse-Geisser correction was applied when sphericity was not met. Analysis included hormone treatments (controls, progesterone, estrogen, and testosterone) as the “between subjects factors” and time as the “within subjects factor” including basal, 2, 4, 6, and 8 hours. A significant (hormone treatment × time) interaction was followed by further breakdown of the analysis by performing one-way RM ANOVA to investigate postprandial ASP changes over time for each hormone treatment independently. Significant differences in ASP levels from basal were reported based on Bonferroni adjusted pairwise comparisons. ASP levels in the analysis were presented as mean differences from basal. For postprandial triglyceride (TG) changes (differences from basal), the area under the curve (TG-AUC) was measured to represent triglyceride clearance. TG-AUC was determined with the linear trapezoidal method calculated by Microsoft Excel Software. Differences in TG clearance between the different hormone treated groups were compared by one-way ANOVA. The correlation of mean ASP levels at specific time points with corresponding TG-AUC for each hormone treated group was presented in a simple scatter graph. Data in this study was analyzed using SPSS 17 statistical software package with significance set at *P* < 0.05.

## 3. Results

### 3.1. Postprandial ASP Level Variations

**Figure 1:**
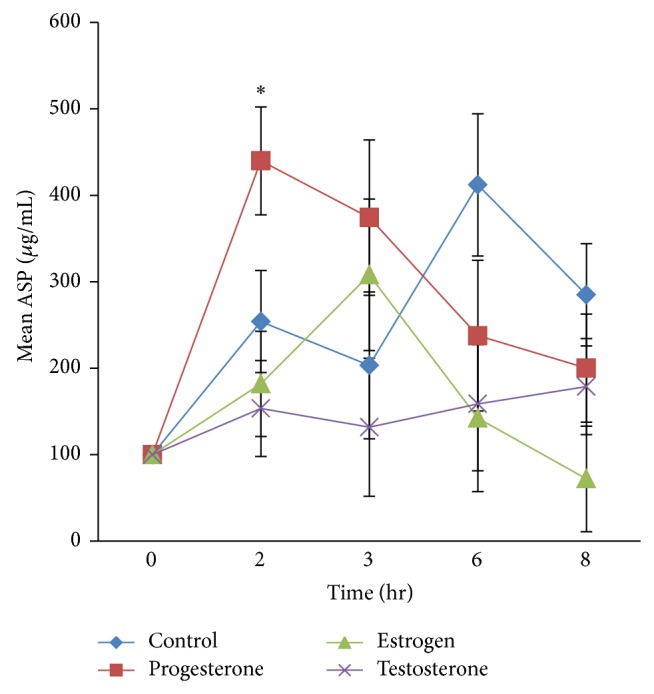
Changes in plasma ASP concentrations after an oral fat load for different hormone treated groups and control group (each group, *n* = 6). Results at each time point during postprandial period are expressed as means ± SEM (*µ*g/mL) of difference in ASP levels from basal (by subtracting basal value at time zero (as shown in Table 2)) from each time point, and percentage increase was calculated. Two-way RM-ANCOVA demonstrated a significant main interaction between hormone treatment and time (hormone treatment × time); *F* = 2.04, *P* = 0.033, and* partial eta squared* = 0.25. Significant changes were set at ^*^ *P* < 0.05, basal ASP levels.

The rat's weights, basal ASP, and TG levels are presented in Table 2. One-way ANOVA showed no significant differences in rat weights at the time of ovariectomies or at the day of fat load administration. In addition, no significant differences in basal ASP or TG levels were detected. Two-way RM-ANCOVA demonstrated a main significant interaction between all hormone treatments and time (hormone treatment × time); *F*_12,72_ = 2.04, *P* = 0.033. This indicated a significant main hormone effect on ASP levels over the postprandial time course. Postprandial ASP level changes are presented in Figure 1. Further breakdown of the analysis by one-way RM ANOVA showed that ASP postprandial changes in the control group, which appeared as a biphasic increase across the postprandial time course, did not reach statistical significance (*P* = 0.10). Similarly, ASP levels in the estrogen and testosterone treated groups across the postprandial course were not significant, and therefore no further analysis was warranted in the repeated measures design. Only the progesterone treated group showed a significant ASP postprandial increase over basal levels at 2 hours (*P* = 0.012), and when compared separately to the controls by 2-way RM ANCOVA, ASP levels in the progesterone treated group were markedly higher than the controls at 2 hours (439.8 ± 62.4 versus 253.45 ± 59.03), *F*_4, 32_ = 2.8, *P* = 0.04,* partial eta squared* = 0.26. ASP levels gradually decreased at 4 and 6 hours and were back to basal levels at 8 hours. Furthermore, ASP level differences at each postprandial time point were compared for the different hormone treated groups by one-way ANOVA. Post hoc comparisons showed that the greatest difference in ASP levels was between the progesterone and testosterone treated groups at 2 hours, where ASP levels in the progesterone treated group was about 3-fold ASP levels in the testosterone treated group (Figure 3(a)).

### 3.2. Postprandial TG Level Variations

**Figure 2:**
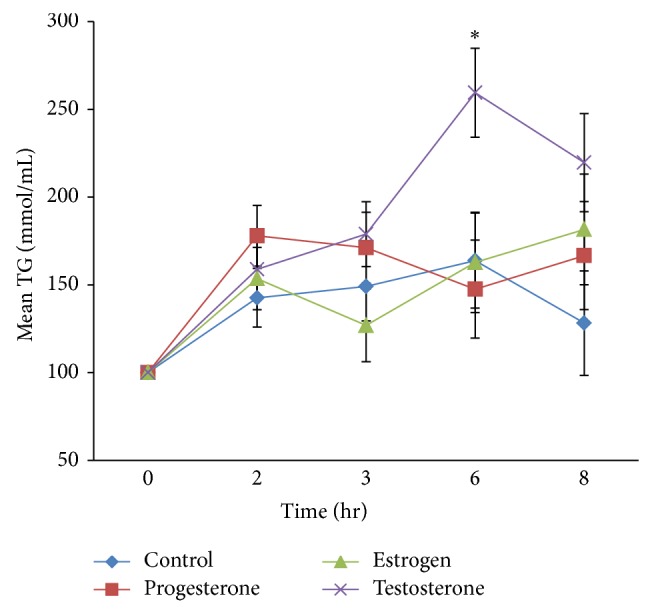
Changes in plasma triglyceride (TG) concentrations for different hormone treated groups and control group (*n* = 6). Results at each time point during postprandial period are expressed as means ± SEM (mmol/L) of difference in TG levels from basal (by subtracting basal values) from each time point and percentage increase was calculated. Two-way RM-ANCOVA adjusting for weight as a covariate demonstrated a significant interaction between hormone treatment and time (hormone treatment × time); *F* = 2.31, *P* = 0.015, and* partial eta squared* = 0.28. Significant changes were set at ^*^ *P* < 0.05.

**Figure 3:**
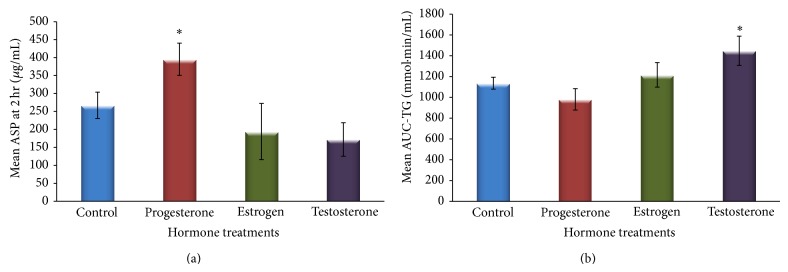
(a) ASP levels at 2 hrs showed that the greatest difference in ASP levels was between the progesterone treated group and the testosterone treated group (*P* = 0.049). (b) Areas under the curves (AUCs) for postprandial changes in plasma triglyceride (TG) concentrations for the different hormone treated groups and control group were measured to represent postprandial TG clearance after a fat load. The greatest difference in TG-AUC was greatest between the progesterone treated group and the testosterone treated group (0.041). Results are expressed as mean ± SEM. Significant differences were set at ^*^ *P* < 0.05.

On the other hand, postprandial TG levels over the time course are shown in Figure 2. Two-way RM-ANOVA showed significant (hormone × time) interactions across the postprandial course between the different groups reflecting a main effect of hormone treatment on postprandial TG levels over time. However, in this study, the main focus was on “TG clearance” which was represented by measuring TG-AUC. One-way ANOVA analysis showed a main significant difference in TG-AUC values between the groups, *F* = 3.17, *P* = 0.047 (Figure 3(b)). Similar to findings on ASP levels at 2 hours, post hoc comparisons of TG-AUC between the different hormone treatments showed that the greatest TG-AUC difference was between the progesterone and testosterone treated groups. However, the trend was opposite to the results seen for ASP levels at 2 hours, as TG-AUC in the progesterone treated group was significantly lower compared to the testosterone treated group representing increased TG clearance in the progesterone treated group compared to the testosterone treated groups that displayed delayed clearance.

### 3.3. Relation of ASP Levels in the Hormone Treated Groups with Corresponding TG Clearance

**Figure 4:**
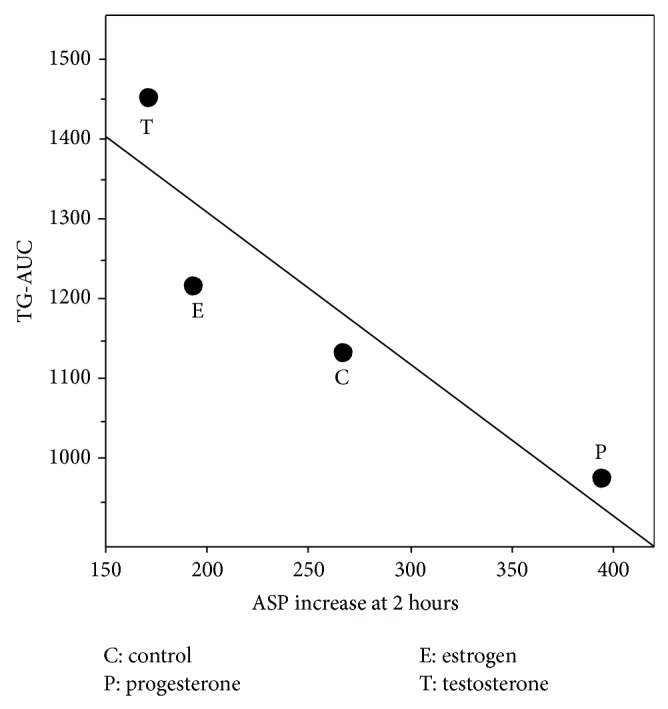
A simple scatter graph representing a negative association between mean postprandial ASP level increase at 2 hours and corresponding mean TG-AUC of the different hormone treated groups.

The opposite trend seen for the ASP levels at 2 hours and corresponding TG-AUC for the different hormone treatments (Figures 3(a) and 3(b)) are further displayed in a scatter graph demonstrating a strong negative association between the means of ASP levels at 2 hours and corresponding TG-AUC for the different hormone treatments (Figure 4).

## 4. Discussion

Postprandial plasma ASP variations after a fat load were shown in several studies [1, 2, 8, 27–29]. One study on humans showed direct postprandial ASP changes* in vivo* by measuring venoarterial gradients across the subcutaneous adipose tissue bed after a fat load. In that study, local ASP production from human adipose tissue was accentuated postprandially after the fat load, and the maximal generation of ASP coordinated with maximal removal of TG [27]. In studies on mice, exogenous ASP treatment was associated with enhanced TG clearance [29]. On the other hand, ASP knockout mice demonstrated delayed TG clearance, which was more pronounced in males, suggesting gender differences in ASP mediated postprandial TG clearance [2]. Evidence that links sex hormones to ASP variations is limited and mainly consists of results that show significant associations between plasma ASP and circulating female hormone levels in humans [13, 15, 30]. Direct effects of female hormones on ASP production and function were shown* in vitro* [31]. However,* in vivo* evidence identifying a direct role for sex hormones in plasma ASP variations is lacking. In this study, initially, acute hormone treatments after one hour had no significant effect on baseline ASP levels. Interestingly however, after the fat load, there was a significant main hormone effect on postprandial ASP levels over time. Postanalysis showed that the main hormone effect was due to progesterone treatment, as there was no significant increase in postprandial ASP levels in the controls or with estrogen and testosterone treatments. The key finding of this study was the effect of progesterone treatment on postprandial ASP levels. The progesterone treated group showed a marked surge in ASP levels at 2 hours which significantly contributed to around 26% variance in postprandial ASP levels (determined by partial eta squared). These findings are interesting especially in connection to a recent study showing a strong association of endogenous ASP fluctuations during the menstrual cycle with progesterone levels in females of the reproductive age. The study showed that ASP levels followed the same pattern of increasing progesterone levels from the follicular towards the luteal phase where ASP levels became significantly elevated [13]. Furthermore, a recent study showed that circulating progesterone levels correlated positively with complement C3 (ASP precursor) gene expression in adipose tissue [30]. Another human study showed that hormone replacement treatment in postmenopausal women increased circulating C3 levels; however, differential effects of female hormones were not determined [32]. In this study, we provide evidence consistent with a direct role for progesterone in enhancing endogenous ASP production* in vivo*.

It is already established that ASP production from adipocytes is largely enhanced by TG-rich chylomicron treatment* in vitro* [33]. After a fat load,* in vivo* TG-rich chylomicrons are highly abundant and in combination with progesterone treatment may contribute to even more ASP production leading to increased systemic ASP levels. Furthermore, the opposite trends shown considering increased postprandial ASP levels and the corresponding change in TG levels in the different hormone treated groups (Figures 1 and 2) and the negative association of ASP levels with TG-AUC (Figures 3 and 4) are indicative of enhanced TG clearance, concurrent with increased ASP levels. This was mostly evident in the progesterone treated group. Conversely, lower ASP levels in the testosterone treated group were associated with delayed TG clearance (presented as increased AUC) which is consistent with evidence in the literature presenting testosterone as an antilipogenic factor [34]. The findings altogether suggest that ASP mediated TG clearance is subject to hormonal influence.

In conclusion, although progesterone is recognized as a lipogenic hormone [35], the mechanisms by which progesterone exerts its function in adipose tissue are poorly understood. Studies have suggested that progesterone stimulates fat clearance and storage by enhancing lipoprotein lipase activity [36] or increasing the expression of transcription factors that control synthesis of fatty acid synthase [37]. Several studies reported that progesterone has antiglucocorticoid effects leading to enhanced subcutaneous fat storage and reduced abdominal fat storage [36, 38]. The findings of this study suggest a causal association between hormone treatment and endogenous ASP production. Enhanced TG clearance in association with increased postprandial ASP levels in the progesterone treated group support the notion of a stimulatory role for progesterone in ASP mediated TG clearance.

The young age of the rats provided further advantage to this study as endocrine and tissue changes that occur with aging, coupled with estrogen treatment, were suggested to place ovariectomized rats at risk for adverse metabolic consequences [39].

To our knowledge, this is the first functional study* in vivo* that demonstrates a cause-effect relationship between acute hormone treatment and endogenous ASP levels* in vivo*. In light of the potent lipogenic role of ASP, this study may contribute to further understanding of mechanisms by which progesterone exerts its lipogenic effects in females.

## Figures and Tables

**Table 1:** Hormones concentrations delivered in 0.2 mL sesame oil.

Treatment	*n*	Dose
Control-OVX	6	0.2 mL oil
Progesterone-OVX	6	4 mg/0.2 mL oil
17-*β* estradiol-OVX	6	5 *µ*g/0.2 mL oil
Progesterone and 17-*β* estradiol-OVX	6	5 *µ*g and 4 mg/0.2 mL oil
Testosterone propionate	6	20 *µ*g/0.2 mL oil

**Table 2:** Mean weight and age of rats (*n* = 6) and basal ASP (*µ*g/mL) and TG (mmol*·*mL) levels after hormone treatment.

	Age (weeks)	Weight (g)	ASP (*µ*g/mL)	TG (mmol/L)
Control	10	214.6 ± 4.3	33.7 ± 4.9	0.74 ± .048
Progesterone	10	211.7 ± 4.8	32.3 ± 2.6	0.63 ± .067
Estrogen	10	195.1 ± 3.9	82.9 ± 22.2	0.55 ± .028
Testosterone	10	205.8 ± 6.4	85.2 ± 25.1	0.59 ± .039

One-way ANOVA, followed by post hoc analysis, showed no significant differences in rat weights at the time of OVX and at the day of the experiment. In addition, no significant differences for basal ASP or TG levels were detected before the experiment (*P* < 0.05).
